# A rapid low-cost real-time PCR for the detection of *klebsiella pneumonia* carbapenemase genes

**DOI:** 10.1186/1476-0711-11-9

**Published:** 2012-06-18

**Authors:** Lijun Wang, Haitong Gu, Xinxin Lu

**Affiliations:** 1 Department of Laboratory Medicine, Beijing Tongren Hospital, Capital Medical University, No1. Dongjiaominxiang Road, Dongcheng District, Beijing, 100730, China

**Keywords:** Real-time polymerase chain reaction, Klebsiella pneumonia carbapenemase

## Abstract

**Background:**

*Klebsiella pneumonia* carbapenemases (KPCs) are able to hydrolyze the carbapenems, which cause many bacteria resistance to multiple classes of antibiotics, so the rapid dissemination of KPCs is worrisome. Laboratory identification of KPCs-harboring clinical isolates would be a key to limit the spread of the bacteria. This study would evaluate a rapid low-cost real-time PCR assay to detect KPCs.

**Methods:**

Real-time PCR assay based on SYBR GreenIwas designed to amplify a 106bp product of the *bla*_KPC_ gene from the159 clinical Gram-negative isolates resistant to several classes of -lactam antibiotics through antimicrobial susceptibility testing. We confirmed the results of real-time PCR assay by the conventional PCR-sequencing. At the same time, KPCs of these clinical isolates were detected by the modified Hodge test (MHT). Then we compared the results of real-time PCR assay with those of MHT from the sensitivity and specificity. Moreover, we evaluated the sensitivity of the real-time PCR assay.

**Results:**

The sensitivity and specificity of the results of the real-time PCR assay compared with those of MHT was 29/29(100%) and 130/130(100%), respectively. The results of the real-time PCR and the MHT were strongly consistent (Exact Sig. (2-tailed) =1. 000; McNemar test). The real-time PCR detection limit was about 0.8cfu using clinical isolates.

**Conclusion:**

The real-time PCR assay could rapidly and accurately detect KPCs -harboring strains with high analytical sensitivity and specificity.

## Background

Carbapenems are widely used to treat serious infections caused by multi-resistant Gram-negative bacteria. However, beginning with the initial description of a novel KPC from an isolate of *K. pneumoniae* in 2001 [1], carbapenem resistance in *Enterobacteriaceae* has been rapidly increasing. KPCs are able to hydrolyze the carbapenems, and cause resistance to multiple classes of antibiotics. Treatment of KPC-producing bacterial infection is thus a considerable challenge for clinicians. KPCs have been reported worldwide, such as North America, South America, Greece, Israel, Puerto Rico, China and so on [2-6]. The expanding geographic spread of KPCs underscores the importance of clinical recognition of these enzymes. In addition, KPCs have been found in bacteria other than *K. pneumoniae,* including *K. oxytoca*[7,8], *P. mirabilis*[9], *Acinetobacter spp*[6]*, P. aeruginosa , C. freundii*[10], *S. marcescens* and *E. coli*[11]. This rapid dissemination of KPC is worrisome. Laboratory identification of KPC-harboring clinical isolates will be critical for limiting the spread.

However, detection of KPC -harboring stains in the clinical laboratory remained a difficult task. The failure of automated susceptibility testing systems to detect KPC-mediated carbapenems resistance was previously reported [12-14]. In 2009, the Clinical Laboratory Standards Institute (CLSI) guidelines (M100) recommended MHT to detect carbapenemase production. Wang *et al*. [15] noted that false positive results could occur when the MHT was used to detect carbapenemase in ESBL-producing isolates. MHT is time-consuming and not routinely tested for *E. cloacae, P. aeruginosa* in laboratory, so that many molecular detection of *bla*_KPC_ genes were evaluated [16-19]. Rapid and sensitive *bla*_KPC_ assays are critical to control the spread of *bla*_KPC_-harboring bacteria in hospitalized patients.

In this paper, we would describe the development of a low-cost real-time PCR assay to screen clinical isolates for *bla*_KPC_.

## Materials and methods

### Bacterial strains

The following reference bacterial strains were the negative controls of *bla*_KPC_: *K. pneumoniae*ATCC13883, extended-spectrum-b-lactamase-positive *K. pneumoniae*ATCC 700603*, A. baumanii*ATCC 19606, *P. aeruginasa*ATCC 27853, *C. albicansATCC* 90029, *E. coliATCC25922*, *E. faecium*ATCC 35667 and *methicillin-resistant S. aureus*ATCC 43300.

The 159 clinical isolates including *K. pneumoniae, E. coli, E. cloacae, K. oxytoca, S. marcescens, P. mirabilis, MDR A.baumanii and* MDR *P. aeruginasa.* were recovered from multiple infection sites like blood, wound, sputum, catheter, urine and pleural effusion from Beijing Tongren Hospital. These clinical isolates were resistant to several classes of -lactam antibiotics, which were identified by antimicrobial susceptibility testing.

### Antimicrobial susceptibility testing

Antimicrobial susceptibility testing was performed with the Vitek 2 susceptibility card AST-GN13 by Vitek 2 automated system (BioMrieux Inc, Durham, NC) according to the manufacturers instructions. Minimum inhibitory concentration (MIC) results of imipenem and ertapenem were classified as susceptible, intermediate, or resistant based on the 2010 CLSI breakpoints (susceptible, 1g/ml and0.25g/ml; intermediate, 2g/ml and 0.5g/ml; resistant, 4g/ml and1g/ml, respectively). However, the AST-GN13 card cannot classify organisms as susceptible to ertapenem without the dilutions less than 0.5g/ml. All clinical isolates were subsequently tested by MHT. The indicator strains in MHT were *E. coli* ATCC 25922 for *Enterobacteriaceae* and *K.pneumoniae* ATCC 700603 for non- *Enterobacteriaceae like P.aeruginosa*[20].

### DNA isolation

Bacterial strains were grown on MacConkey agar and incubated overnight at 35C. One colony was resuspended in 100l of sterile distilled water and the cells were lysed by heating at 100C for 10min. Cellular debris was removed by centrifugation at 13000g for 10min, and the supernatant was used as a source of template DNA for amplification.

For analytical sensitivity based on bacterial colony-forming unit (cfu), DNA isolation was performed using the DNeasy Blood&Tissue Kit (Qiagen Sciences, Maryland, USA) according to the protocol suggested by the manufacture. In brief, a bacterial suspension equivalent to that of a 2.0 McFarland standard was prepared in saline, then 200l (8.010^7^cfu ) suspension were serially diluted 10-fold in saline. Bacterial total nucleic acid was extracted from 200l of each dilution and then eluted in 50l elution buffer and stored at 20C.

### *bla*_KPC_ detection by PCR sequencing

The presence of *bla*_KPC_ was confirmed by conventional PCR and sequencing [1]. The primers included the forward (5-TGTCACTGTATCGCCGTC-3) and the reverse (5- CTCAGTGCTCTACAGAAAACC-3) , The PCR reaction system contained 0.5M each primer, 2Easy*Taq* PCR SuperMix (TransGen Biotech, Beijing, China) and 2l DNA template. The reactions were amplified in a My Cycler thermal cycler (BIO-RAD, USA). Cycling parameters were 5min at 95C, followed by 35 cycles of 1min at 95C, 30s at 58C, and 1min 30s at 72C. The PCR amplification was ended by a final extension at 72C for 10min. sequencing of the PCR products was commercially performed by SinoGenoMax Co. Ltd (Beijing, China). For sequence analysis, the BLAST program from the National Center for Biotechnology Information Web site was used (http: //www. ncbi. nlm. nih.gov/BLAST).

### *bla*_KPC_ detection by real-time PCR

The forward primer sequence (5-TTGTTGATTGGCTAAAGGG-3) and reverse primer sequence (5- CCATACACTCCGCAGGTT-3) were designed in the conservative region of several *bla*_KPC_ types (*bla*_KPC 2_ to *bla*_KPC-13_). The *bla*_KPC_ amplicon was 106 base pairs (GenBank: EU244644).

The 25l real-time PCR mixture contained 12. 5l TransStart Green qPCR super MIX (TransGen Biotech, Beijing, China), 0.5l PCR enhancer (TransGen Biotech, Beijing, China), 0.2M each primer, 9l sterile distilled water and 2l DNA template. Real-time PCR amplification was performed using the Roche Light cycler 480 Real-time system (Roche Diagnostics, Mannheim, Germany). Cycling parameters were 5min at 95C, followed by 40 cycles of 15s at 95C, 15s at 55C, and 30s at 72C. Single fluorescence detection was performed in each cycle at 55C. Melting curve acquisitions were done immediately after the final amplification step by heating at 96C for 5s, cooling to 55C for 1min, and heating slowly at 0.11C per second to 96C with continuous fluorescence recording. Melting curves were recorded by plotting fluorescence signal intensity versus temperature. Amplicon melting temperatures(Tm) were determined by calculating the derivative of the curve using Roche Light cycler 480 software. The results were visualized by plotting the negative derivative against temperature.

### Specificity and sensitivity

In order to determine analytical sensitivity of our assay, *bla*_KPC_ real-time PCR experiments were performed on 10-fold serial dilutions of bacterial cultures (8.010^7^cfu). To evaluate the analytical specificity, a panel of reference stains and clinical strains resistant to several classes of -lactam antibiotics was tested. For statistical analysis, we used the MHT as the reference standard. The differences between sensitivities of the real-time PCR assay and MHT were evaluated with the McNemar test.

## Results

The specificities of the real-time PCR primers for the detection of *bla*_KPC_ genes were evaluated by the BLAST search program, available at www.ncbi.nlm.nih.gov.

The *bla*_KPC_ real-time PCR assay was negative with DNA extracted from the following reference bacterial isolates: *K*.pneumoniae ATCC 13883, extended-spectrum-b-lactamase-positive *K*.pneumoniae ATCC 700603*, A. baumanii* ATCC 19606, *P. aeruginasa* ATCC 27853, *C. albicans* ATCC 90029, *E. coli. ATCC25922*, *E. faecium*ATCC 35667 and *methicillin-resistant S. aureus*ATCC 43300*.*

159 clinical isolates were categorized according to the susceptibility of imipenem or ertapenem (Table1). 53 isolates were intermediate to resistant to imipenem or ertapenem, and 106 isolates were susceptible to carbapenems. 29 clinical isolates with carbapenem resistance or decreased susceptibility were positive by MHT and by real-time PCR and sequencing, respectively. The 29 clinical isolates included 20*K. pneumoniae* (MICs: imipenem16g/ml, ertapenem8g/ml), 3 *E. coli* (one strain MICs: imipenem1g/ml, ertapenem=2g/ml; two strains MICs: imipenem16g/ml, ertapenem8g/ml), 4 *E. cloacae* (MICs: imipenem16g/ml, ertapenem8g/ml), one *S. marcescens* (MICs: imipenem16g/ml, ertapenem8g/ml), and one MDR *A. baumanii* (MICs: imipenem16g/ml, ertapenem8g/ml). One carbapenem-resistant isolate of *K. pneumoniae* (MICs: imipenem16g/ml, ertapenem8g/ml) recovered from sputum was MHT ( ) / *bla*_KPC_ ( ) in our assay. In addition, both isolate of *K. pneumonia* (MICs: imipenem1g/ml, ertapenem8g/ml) and *E. coli* (MICs: imipenem1g/ml, ertapenem=2g/ml) were MHT ( ) / *bla*_KPC_ ( ), which were recovered from catheter. All 130 isolates (33*K. pneumoniae*, 42 *E. coli*, 15 *E. cloacae*, 10*K. oxytoca*, one *S. marcescens*, 8 *P. mirabilis*, 10 MDR *A. baumanii* and 11 MDR *P. aeruginasa* ) were MHT ( ) / *bla*_KPC_ ( ) (Table2). The sensitivity of the real-time PCR assay as compared to the MHT was 29/29(100%) with a specificity of 130/130 (100%). The results of the real-time PCR and the MHT were strongly consistent (Exact Sig. (2-tailed) =1.000; McNemar test).

**Table 1:** Results of Carbapenem susceptibility and MHT of clinical isolates

Species	No. of isolates				
		Intermediate to resistant	Susceptible	Positive	Negative
*K. pneumoniae*	53	22	31	20	33
*E. coli*	45	4	41	3	42
*E. cloacae*	19	4	15	4	15
*K. oxytoca*	10	0	10	0	10
*P. mirabilis*	8	0	8	0	8
*S. marcescens*	2	1	1	1	1
*MDR A. baumanii*	11	11	0	1	10
MDR *P. aeruginasa*	11	11	0	0	11

*: MHT modified Hodge test.

**Table 2:** *bla*_KPC_ Real-time-PCR results compared with the MHT.

*bla*_KPC_ Real- time-PCR*		
	Positive	Negative
Positive	29	0
Negative	0	130

^*^: The percent sensitivity was 100%, and the percent specificity was 100%.

Exact Sig. (2-tailed) =1.000; McNemar test.

All *bla*_KPC_ genes of KPC-producing isolates in this study were verified as *bla*_KPC-2_ by sequencing assay.

**Figure 1:**
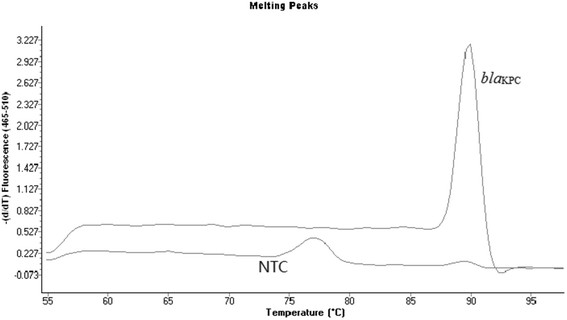
**Plot of the negative derivative of the melting curves vs. temperature: peak indicates the Tm (about 89C) of isolates**. NTC: no template control.

The *bla*_KPC_ amplicon was distinguished by its specific Tm value. Under our experimental conditions, analysis of the melting curve profile of the PCR products indicated that the products peaked at about 89C (Figure1).

**Figure 2:**
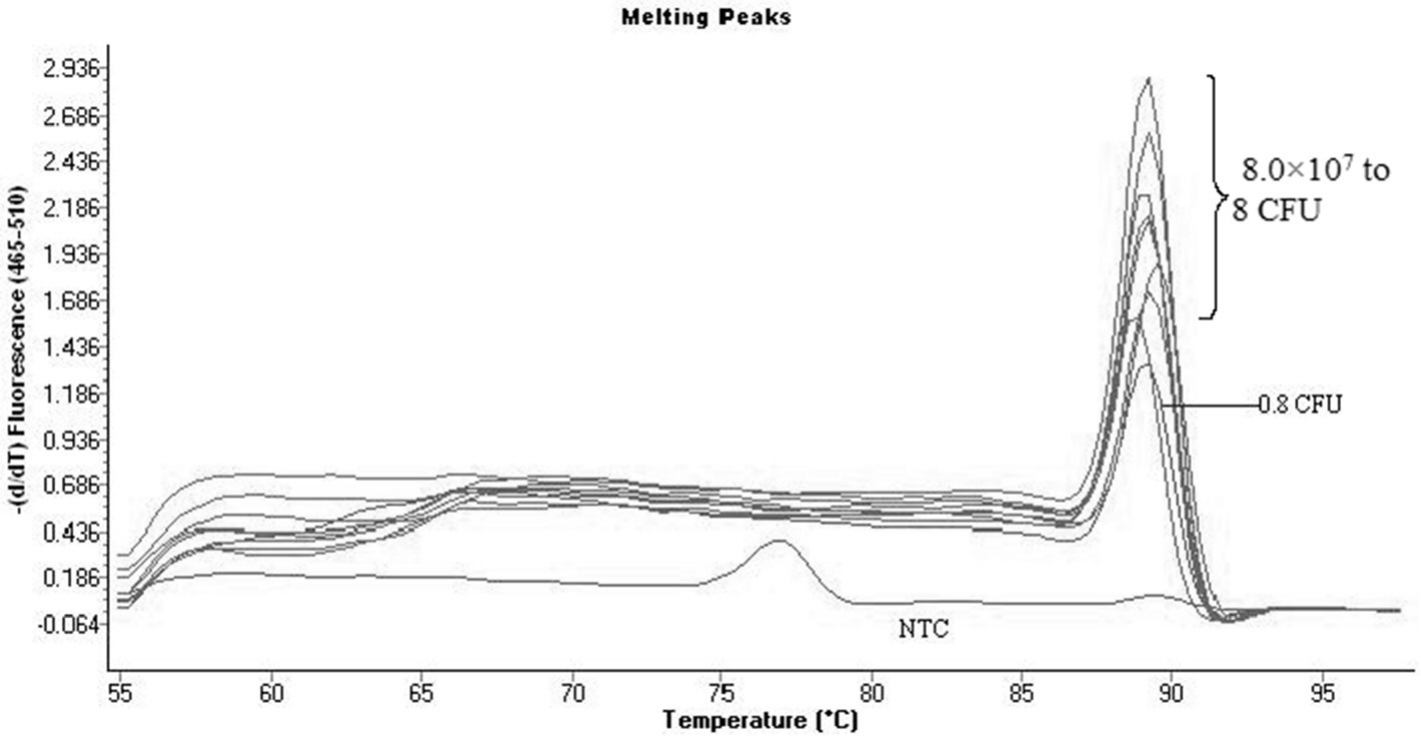
**Analytical sensitivity of the*bla***_**KPC**_**real-time-PCR assay showing a minimum detection limit of 0.8CFU**.

The analytical sensitivity of the *bla*_KPC_ real-time PCR assay was determined after serially diluting known concentrations (8.010^7^cfu) of clinical isolated carbapenems-resisant *K. pneumoniae*. The dynamic range of the assay covered nine orders of magnitude from 8.010^7^ to 0.8cfu. *bla*_KPC_ specific fluorescent peaks were detected in the isolates dilutions to about 0.8cfu (Figure2).

## Discussion

Along with the wide use of carbapenem antibiotics, KPCs appeared a major public health concern. Bacterial isolates producing KPCs are able to hydrolyze a broad spectrum of -lactams including the penicillins, cephalosporins, carbapenems and monobactam. They have the potential to spread rapidly in hospital environments to cause nosocomial infections with high mortality rates [21]. KPC-producing *Enterobacteriaceae* stains are increasingly spreading throughout China [2,9,11,22]. The dominant clone of KPC-producing *K. pneumoniae* in China is ST11, which is closely related to ST258 reported worldwide [23]. A rapid method confirming KPCs is significant to control this spread.

In 2009, the CLSI recommended MHT to screen for the production of carbapenemase in *Enterobacteriaceae* isolates with elevated MICs for carbapenems or reduced inhibition zones measured by disc diffusion. In 2010, Carbapenem breakpoints have changed in M100-S20U and M100-S21 with 2-fold lower MICs of each category (susceptible, intermediate, and resistant) for the *Enterobacteriaceae* for imipenem, meropenem, and ertapenem. According to the new criteria, the initial screen test and the confirmatory test by MHT are no longer necessary for routine patient testing. However, one isolate of *E. coli* with MIC to imipenem as low as 1g/ml was confirmed as MHT ( + ) / *bla*_KPC_ ( + ) in our study. Decreased ertapenem susceptibility has been considered as one of the most sensitive phenotypic indicators of KPC production, but it has been found to be nonspecific [24,25]. In our laboratory, two clinical isolates MICs to ertapenem as high as 2g/ml to 8g/ml were MHT ( ) / *bla*_KPC_ ( ). Despite CLSI new recommendations, our laboratory continued to confirm KPC using MHT or PCR.

The sensitivity and specificity of the MHT have been shown to exceed 90%; however, several reports have noted false positive results occurred when the MHT was used to detect carbapenemase in ESBL-producing isolates [15,26]. In addition, it may not be the ideal phenotypic confirmatory test for KPCs since interpretation can be difficult for some isolates such as *A. baumanii, P. aeruginasa*. In our study, we adjusted the indicator stain to *K. pneumoniae*ATCC 700603 for non- *Enterobacteriaceae* in order to eliminate the incidence of indeterminate results of MHT [20]. Thus, an alternative method may prove to be more useful. During the recent few years, molecular methods have been used to rapidly detect *bla*_KPC_ genes. In particular, real-time PCR assays offered the advantage of shorter turnaround time, which were even developed to detect KPC-containing strains with high analytical specificity and sensitivity in surveillance specimens [27,28].

In this study, we validated a rapid, sensitive, and specific real-time-PCR assay for the detection of *bla*_KPC_ genes. This assay can be performed in less than 4 hours, which will reduce the chance of spreading the organism in the hospital. The real-time PCR assay specifity and sensitivity were 100% compared to phenotypic KPC activity assessed by MHT and sequencing. Thirteen KPC gene variants have been described, classified in sequential numeric order from *bla*_KPC-1/2_ to *bla*_KPC-13_. The *bla*_KPC_ genes are characterized by nonsynonymous single nucleotide substitutions [17]. Our sequencing results showed all 29 KPC-producing isolates harbored *bla*_KPC-2_ gene. KPC-2 clinical isolates were widely isolated in most parts of China [23,29]. Last year, Li et al [30] in China firstly described KPC-3-harboring E. coli and C. *freundii*. Although KPC-2 and KPC-3 were well described throughout China, we designed the primers in conservative areas to ensure that our assay could almost detect the variants currently described. We identified *bla*_KPC_ genes by melting curve analysis of the amplification product using SYBR GreenIwith many advantages like low-cost and easy to use. The Tm value of the *bla*_KPC_ gene was detected at about 89C. Our assay sensitivity is about one cfu sufficient to detect *bla*_KPC_containing isolates.

## Conclusions

The real-time PCR assay described here provides a useful screening test to detect *bla*_KPC_ genes rapidly and accurately. Although the real-time PCR assay was unable to identify the specific gene in the *bla*_KPC_ family in clinical isolates, accurate and rapid identification of this kind of resistance genes is the first step to control their spread.

## Abbreviations

KPC = Klebsiella pneumonia carbapenemase; MHT = modified Hodge test; CLSI = the Clinical Laboratory Standards Institute; cfu = colony forming unit; MIC = Minimum inhibitory concentration.

## Competing interests

The authors declare that they have no conflict of interest.

## Authors' contributions

LJ Wang carried out the real-time PCR studies, participated in the sequence alignment and drafted the manuscript. HT Gu carried out the bacterium isolation and antibiotic sensitivity test. XX Lu participated in the design of the study and coordination and helped to draft the manuscript. All authors read and approved the final manuscript.
